# Depressive symptoms in people with late effects of polio and the association with sociodemographic and disability‐related factors

**DOI:** 10.1002/pmrj.70049

**Published:** 2025-11-11

**Authors:** Maria Nolvi, Christina Brogårdh, Lars Jacobsson, Jan Lexell

**Affiliations:** ^1^ Department of Health Sciences Lund University Lund Sweden; ^2^ Department of Neurology and Rehabilitation Medicine Skåne University Hospital Lund Sweden; ^3^ Department of Rehabilitation Sunderby Hospital Luleå Sweden

## Abstract

**Background:**

Late effects of polio (LEoP) is a progressive condition leading to a lifelong disability that can affect mental health. There is limited knowledge of depressive symptoms and associated factors in people with LEoP.

**Objective:**

To assess the occurrence of depressive symptoms in people with LEoP and explore the association with sociodemographic and disability‐related factors.

**Design:**

Cross‐sectional survey.

**Setting:**

University hospital outpatient clinic.

**Study participants:**

Eighty‐one people (mean age 73 years, 49% women) with LEoP.

**Main outcome measurements:**

Swedish versions of the 20‐item Geriatric Depression Scale (GDS‐20), the Self‐reported Impairments in Persons with late effects of Polio (SIPP) scale, and the Reintegration to Normal Living Index (RNL‐I).

**Methods:**

The participants responded to a postal survey including the GDS‐20 and questionnaires about sociodemographic factors (gender, age, marital status) and disability‐related factors (SIPP, mobility, RNL‐I). To determine factors associated with depressive symptoms (GDS‐20, dependent variable), univariable and two separate multivariable logistic regression models were created, comprising sociodemographic and disability‐related factors, respectively.

**Results:**

Thirty‐five people (43%) had a GDS score of six points or more, indicating suspected depression. In the model with sociodemographic factors, only marital status was significant (*p* = .001) with an odds ratio (OR) of 5.53 (95% confidence interval [CI], 1.97–15.54). In the model with disability‐related factors, self‐reported impairments and perceived participation remained significant, with self‐reported impairments having the highest OR (OR, 1.49; 95% CI, 1.18–1.88). Overall, disability‐related factors had a larger explanatory value (Nagelkerke *R* ^2^ 0.73) for suspected depression than sociodemographic factors (Nagelkerke *R* ^2^ 0.24).

**Conclusion:**

The relatively high occurrence of suspected depression in people with LEoP implies that screening for depression is important. It remains to be determined if rehabilitation interventions targeting disability‐related factors can affect mental health in people with LEoP.

## INTRODUCTION

People who had paralytic polio in their childhood can decades later experience new symptoms such as muscle weakness and fatigue, muscle and joint pain, general fatigue, sleep disturbances and concentration difficulties,^1^ referred to as postpolio syndrome or late effects of polio (LEoP). The condition is progressive and leads to a lifelong disability, which can affect the person's performance of daily activities and their perceived participation. This altered life situation can, in turn, affect their mental health and develop into a depression.

Depression is a mental illness that is characterized by a low mood. Other symptoms such as lack of energy, concentration difficulties, and sleep disturbances occur frequently. Having a depressive disorder means great suffering for the person, but it can in many cases be successfully treated. It is known that people with disabilities have an increased risk for depression, with a prevalence of 25%–50%.^2^ In survivors of polio, studies have shown varying results, ranging from normal levels^3^, ^4^, ^5^ to a suspected depression in >40% of the investigated population.^6^ The different results can be due to several factors such as the use of various rating scales and different sociodemographic factors of the populations studied. Gender, age, and marital status can be important factors for the development of depressive symptoms, as studies have found elevated depressive symptoms in women, elderly people,^7^ and people experiencing loneliness^7^ combined with isolation.^8^

Also, LEoP‐related impairments and the severity of the disability could be of importance. A previous study found that motor function was not associated with depressive symptoms,^9^ but no study has investigated the association between common LEoP‐related impairments and depressive symptoms. Finally, the impact of the disability on activities and participation could be of importance for the occurrence of depressive symptoms. In other disability groups,^10^, ^11^, ^12^ higher levels of activity and perceived participation were associated with fewer depressive symptoms. Taken together, there is limited knowledge of the occurrence of depressive symptoms in people with LEoP and the association with factors that could contribute to depressive symptoms. Such knowledge could raise awareness of depressive symptoms in people with LEoP and assist in the development of rehabilitation interventions supporting mental health.

Thus, the aim of this study was to assess the occurrence of depressive symptoms in people with LEoP and to explore the associations with sociodemographic factors (gender, age, and marital status) and disability‐related factors (self‐reported impairments, mobility, and perceived participation).

## MATERIALS AND METHODS

### Study participants

The participants were selected from a larger project, “Living with Late Effects of Polio from a Salutogenic Perspective.”^13^ Originally, 130 people had been randomly selected from a cohort of 325 people who had taken part in a rehabilitation program in a clinic in southern Sweden and who met the inclusion criteria of being community dwelling and ambulatory. Of the 130 potential participants, 97 people accepted to participate whereof 81 had responded to the Geriatric Depression Scale (GDS) and thus constituted the final sample.

All participants had verified LEoP with a history of acute polio, a stable period of at least 15 years, and new symptoms after this period. An electromyogram had been recorded in their arms and legs as part of the verification of a history of polio, and all participants had electromyogram findings in agreement with prior polio in at least one limb. The participants had no other comorbidity (such as Parkinson's disease, stroke, or other major condition) that could explain their new symptoms. All participants except one were of Swedish origin.

### Ethical considerations

The principles of the Helsinki Declaration were followed, and the study was approved by the Regional Ethical Review Board in Lund, Sweden (Dnr 2016/134). All participants gave their written informed consent to participate in the study.

### Procedure

A letter with information about the project; a questionnaire about sociodemographics (gender, age, marital status), mobility (walking ability), and use of mobility aids (crutches, rollator, orthoses); the rating scales; an informed consent form; and a prepaid envelope for returning the forms was sent to all potential participants. In the present study the Swedish versions of the following rating scales were used: GDS‐20, Self‐reported Impairments in Persons with Late Effects of Polio (SIPP) scale, and Reintegration to Normal Living Index (RNL‐I).

### Rating scales

To assess the occurrence of depressive symptoms, the GDS‐20 was used.^14^ GDS‐20 consists of 15 questions from the original short form of GDS covering common symptoms of depression such as mood, lack of energy and social withdrawal,^15^ and 5 additional questions covering insomnia, anxiety, panic, pain, and hypochondria. The five additional questions were added to increase the sensitivity of the scale because these symptoms are important signs of depression in older people. The GDS‐20 has been found to be a valid screening instrument for depression in older adults.^14^ The questions are answered with “yes” or “no,” where each question gives 1 point if the answer is a possible sign of depression. A total sum score of ≤5 points is interpreted as depression being unlikely; if the total score is ≥6 points depression should be suspected.

Self‐reported impairments, directly or indirectly related to LEoP, were assessed with the SIPP scale.^16^ The scale consists of 13 items assessing how much the respondent has been bothered by LEoP‐related impairments during the past 2 weeks such as muscle weakness, muscle and/or joint pain during activity and at rest, cold intolerance, and general fatigue. There are four response options, ranging from 1 (not at all) to 4 (extremely). The total sum score ranges from 13 to 52 points; higher scores indicate that the person is more bothered by the impairments. The SIPP scale has been Rasch analyzed and examined for test–retest reliability. It is found to be unidimensional^16^ and has an intraclass correlation coefficient of 0.88.^1^

To assess perceived participation, the RNL‐I was used.^17^ The RNL‐I consists of 11 items covering self‐perceived participation in the life situation with statements such as “I am able to participate in recreational activities as I want to” and “I assume a role in my family which meets my needs and those of other family members.” For each statement there are four response options, ranging from 1 (“does not describe my situation”) to 4 (“fully describes my situation”). The total item score ranges from 11 to 44 points, where greater scores indicate higher participation in life events. A review of the psychometric properties of the RNL‐I, evaluated in people undergoing rehabilitation, showed that it is valid and reliable with Cronbach's *α* values of 0.73–0.97.^18^ In a study of the test–retest reliability of the RNL‐I in people with LEoP, the intraclass correlation coefficient was 0.88.^19^

### Data and analyses

Means, SDs, range, number, and percent values were calculated when appropriate. To examine associations with the occurrence of depressive symptoms, univariable logistic regression analyses were first performed with all selected variables: that is, sociodemographic factors (gender, age, marital status) and disability‐related factors (self‐reported impairments, mobility, perceived participation). For the dichotomous variables, gender and marital status, women and living alone were coded as the reference categories. For mobility, dummy variables were coded for walking ability 100 to 1000 meters and more than 1000 meters, with walking ability less than 100 meters as the reference category. The occurrence of depressive symptoms (dependent variable) was dichotomized into “unlikely depression” (GDS score of ≤5 points) and “suspected depression” (GDS score of ≥6 points). Odds ratios (OR) with 95% confidence intervals (CIs), Nagelkerke *R* ^2^ (explanatory value), and *p* values were calculated in the analyses.

To examine multiple impact on the occurrence of depressive symptoms, multivariable logistic regression analyses were performed with “unlikely depression” versus “suspected depression” (ie, GDS scores of ≤5 points vs. ≥6 points) as the dependent variable. All selected independent variables were included in the multivariable analyses. Because our sample size allowed a maximum of three independent variables per model, two separate multivariable logistic models were created: one model for sociodemographic factors and one model for disability‐related factors.

The IBM SPSS statistics version 28^20^ was used throughout. A *p* < .05 was considered to represent statistical significance.

## RESULTS

### Participant characteristics

The characteristics of the 81 participants are presented in Table 1. There were 40 (49%) women and 41 (51%) men, with a mean age of 73 years (SD 8.3, range 41–89). There was no significant difference between the participants and nonrespondents regarding their gender or age. Forty‐nine participants (60%) were married/cohabiting whereas 29 participants were living alone. Twenty‐four participants (30%) could walk <100 meters, 33 participants (41%) between 100 to 1000 meters, and 24 participants (30%) could walk >1000 meters. A majority (62 participants, 77%) were using mobility aids (wheelchair, crutches, rollator, orthoses).

**TABLE 1:** Characteristics of the 81 participants with late effects of polio and the nonrespondents.

	Participants	Nonrespondents
Gender (*n*, %)		
Men	41 (51)	18 (55)
Women	40 (49)	15 (45)
Age (y)		
Mean (SD)	73.1 (8.3)	72.9 (9.1)
Range	41–89	39–87
Marital status (*n*, %)		
Single	29 (36)	
Married/cohabiting	49 (60)	
Walking ability (*n*, %)		
<100 m	24 (30)	
100–1000 m	33 (41)	
>1000 m	24 (30)	
Use of mobility aids (*n*, %)	62 (77)	
Wheelchair	14 (17)	
Crutches, rollator	45 (56)	
Orthoses	41 (51)	
Number of years with LEoP		
Mean (SD)	21.8 (8.9)	
Range	6–55	

Abbreviation: LEoP, late effects of polio.

### Depressive symptoms

**FIGURE 1:**
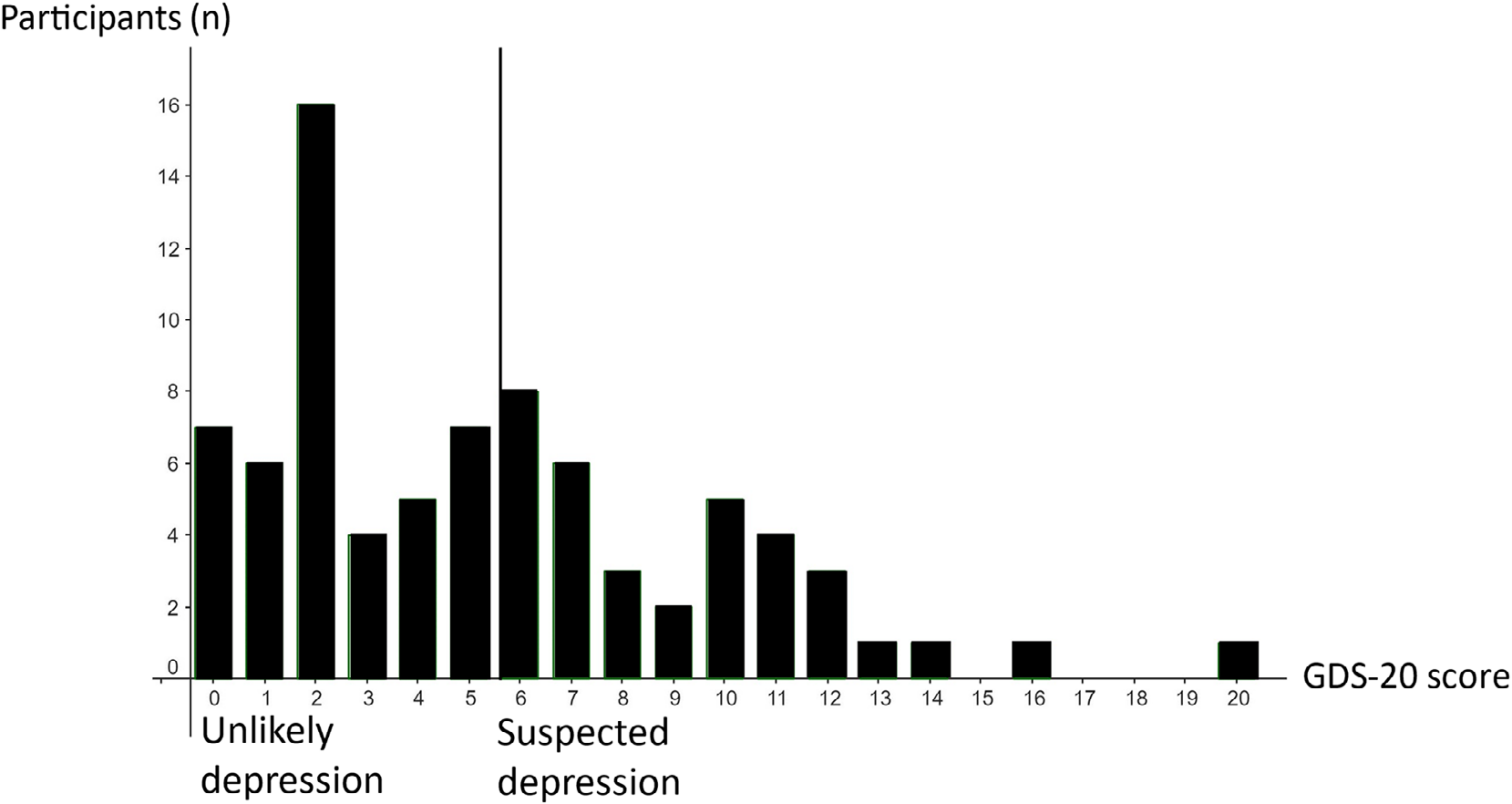
Distribution of the 20‐item GDS‐20 scores in 81 people with late effects of polio. GDS‐20 is dichotomized into unlikely depression (≤5 points) and suspected depression (≥6 points). GDS‐20, Geriatric Depression Scale.

The distribution of the occurrence of depressive symptoms is presented in Figure 1. The mean total GDS‐20 sum score was 5.4 (SD 4.2, median 5). Forty‐six (57%) participants had few depressive symptoms and scored within the range of the GDS‐20 where depression is unlikely. Thirty‐five (43%) participants had a GDS score of ≥6 points, indicating “suspected depression.”

### Self‐reported impairments and perceived participation

The mean SIPP score was 28.4 (SD 7.6) points and the mean RNL‐I score was 35.3 (SD 7.2).

### Univariable logistic regression analyses

The univariable logistic regression analyses are presented in Table 2. Being single, having more self‐reported impairments, and having a walking ability <100 meters were significantly associated with the probability of a GDS score in accordance with “suspected depression.” A high level of perceived participation was significantly associated with a reduced likelihood of a GDS score in accordance with “suspected depression.” Marital status had the largest OR of 5.56 (95% CI, 2.04–15.13, *p* < .001); participants being single were more likely to report suspected depression compared to individuals who were married or cohabitating.

**TABLE 2:** Univariable regression analyses of the independent variables with Geriatric Depression Scale as the dependent variable.

	Distribution (*n*, %)	Suspected depression (*n*, %)	Odds ratio (CI)	*p* value	Nagelkerke *R* ^2^
Gender			1.16 (0.48–2.78)	.75	0.00
Women^a^	40 (49)	18 (45)			
Men	41 (51)	17 (41)			
Age			1.06 (1.00–1.13)	.05	0.07
Marital status			5.56 (2.04–15.13)	<.001	0.20
Single^a^	29 (36)	20 (69)			
Cohabitating	49 (60)	14 (29)			
SIPP			1.37 (1.19–1.58)	<.001	0.56
Mobility			3.08 (1.15–8.29)	.03	0.08
< 100 m^a^	24 (30)	15 (63)			
>100 m	57 (70)	20 (35)			
RNL‐I			0.80 (0.72–0.88)	<.001	0.46

*Note*: GDS‐20 is dichotomized into unlikely depression (≤5 points) and suspected depression (≥6 points).

Abbreviations: CI, confidence interval; RNL‐I, Reintegration to Normal Living Index; SIPP, Self‐reported Impairments in Persons with late effects of Polio scale.

^a^ Reference categories.

### Multivariable logistic regression analyses

The multivariable logistic regression analyses are presented in Table 3. In the model with sociodemographic factors, only marital status remained significant (*p* = .001) with an OR of 5.53 (95% CI, 1.97–15.54). In the model with disability‐related factors, self‐reported impairments and perceived participation remained significant. Self‐reported impairments had the highest OR (OR, 1.49; 95% CI, 1.18–1.88); participants that were more bothered by LEoP‐related impairments were more likely to report suspected depression. Overall, disability‐related factors had a larger explanatory value (Nagelkerke *R* ^2^ 0.73) to suspected depression than sociodemographic factors (Nagelkerke *R* ^2^ 0.24).

**TABLE 3:** Multivariable regression model with Geriatric Depression Scale as the dependent variable.

	Odds ratio (CI)	*p* value	Nagelkerke *R* ^2^
Sociodemographic factors			
Gender, women^a^	1.02 (0.37–2.79)	.97	0.24
Age	1.06 (0.99–1.13)	.08	
Marital status, single^a^	5.53 (1.97–15.54)	.001	
Disability‐related factors			
SIPP	1.49 (1.18–1.88)	<.001	0.73
Mobility <100 m^a^	0.29 (0.03–2.80)	.29	
RNL‐I	0.75 (0.64–0.89)	<.001	

*Note*: GDS‐20 is dichotomized into unlikely depression (≤5 points) and suspected depression (≥6 points).

Abbreviations: CI, confidence interval; GDS‐20, Geriatric Depression Scale; RNL‐I, Reintegration to Normal Living Index; SIPP, Self‐reported Impairments in Persons with late effects of Polio scale.

^a^ Reference categories.

## DISCUSSION

In the present study we found that as many as 43% of the participants with LEoP scored ≥6 points, that is, above the cutoff point of GDS‐20 for when depression should be suspected. The multivariable analyses revealed that being single, having more self‐reported impairments, and having less perceived participation significantly contributed to a GDS score indicating suspected depression.

### Depressive symptoms

We found a relatively high occurrence of depressive symptoms among our participants, and suspected depression was also more common than in earlier studies of survivors of polio where the GDS was used. In a study of 121 survivors of polio, Kemp and coworkers found that 28% had symptoms reflecting depressive disorder when using the GDS‐30.^21^ In a later study, Kemp and Krause found a lower prevalence, with significant depressive symptoms in 20% of the people with LEoP.^22^ Our result, when using the GDS‐20, is close to the prevalence of 41% in survivors of polio (with or without LEoP) found in a study using the GDS‐15.^6^

The occurrence of depressive symptoms in studies using other assessment tools have also varied. Pierini and coworkers reported moderate‐to‐severe depressive symptoms in 40% of their population with LEoP (*n* = 630), assessed with the Center for Epidemiologic Studies Depression Scale.^23^ In a small study of people with LEoP (*n* = 16) and using the Hamilton Depression Scale, Weber and coworkers found depressive episodes in 53% of their participants.^9^ In contrast to these findings, Curtis and coworkers found a very low prevalence of depressive symptoms, as assessed with the Patient Health Questionnaire, in 214 people with LEoP who underwent a self‐management program.^3^ Two earlier studies among polio survivors also found depressive symptoms within the normal range, assessed with the Hospital Anxiety and Depression scale^4^ and the Brief Symptom Inventory.^5^ The different results may be due to the use of various rating scales and definitions of depressive symptoms and depression and also due to various sociodemographic and disability‐related factors of the populations studied.

Although the prevalence of depressive symptoms varies among people with LEoP,^3^, ^4^, ^5^, ^6^, ^9^, ^21^, ^22^, ^23^ it is still higher compared to the general population. In the development of the GDS‐20, Gottfries and coworkers found suspected depression in 9.3% of 1002 elderly people visiting health care centers in Sweden.^14^ In another study of 6659 older people randomly selected from the general population in Sweden, the prevalence of depressive symptoms, assessed with the Hospital Anxiety and Depression scale, was 9.8%.^24^ It may be that some people who scored above the cutoff point of suspected depression in the present study did so because the GDS‐20 contains two questions of symptoms frequently occurring also in LEoP: pain and lack of energy. An older study suggested that the high depression rates in people with LEoP might reflect the populations' early stage in the coping process, and this may partly explain the different results in earlier studies of people with LEoP.^4^ However, this explanation does not apply to our sample, who had been living with LEoP for >2 decades (cf. Table 1).

### Depressive symptoms and sociodemographic factors

In our regression model with sociodemographic factors, marital status was significantly associated with suspected depression: people who were single reported significantly more depressive symptoms compared to those who were married or cohabitating. This is in line with many studies reporting that loneliness, being single, and living alone are associated with depressive symptoms.^5^, ^8^, ^24^, ^25^ In addition, studies have shown that satisfaction with social relations^4^ and social support^26^, ^27^ are associated with fewer depressive symptoms. When living with someone, you can share the joys and burdens of everyday life with that person, and this person can also provide social support. Living with someone also means that you are needed by that person and can provide support yourself, which has been found important for meaningfulness in people with LEoP^28^; this could be a protective factor against depression.

We found no significant association between gender and suspected depression in the regression model. This is in contrast to studies where gender differences were found. In a study of people with LEoP, self‐reported depression was more common in women.^29^ Another study using GDS‐30 found significantly higher depression scores in women with LEoP.^21^ Two studies comprising elderly people in the general population also found more depressive symptoms in women.^7^, ^30^ However, contradictory results were found in a study using the Hospital Anxiety and Depression scale, performed in Sweden of 6659 randomly selected older people from the general population with a mean age of 71 years. In that study, where the prevalence of depressive symptoms and loneliness and the association to age and gender was investigated, depressive symptoms were more prevalent in men.^24^ The authors of that study discussed that men might more often exhibit alternative depressive symptoms not captured by many rating scales, which may explain the differing results.

We also found no significant association between the occurrence of suspected depression and the participants' age. No age difference in depressive symptoms has been described before in people with LEoP.^23^ This is in contrast to the results in a study of older people from the general population in Bangladesh, which found that participants aged 71 and older were more likely to develop depressive symptoms.^7^ A study in Sweden of older people in the general population found a larger prevalence of depressive disorder in men with increasing age, with the highest prevalence among those 75 to 80 years.^24^ The consequences of growing older might include functional deterioration with participation restrictions, loss of loved ones, and loneliness, which may contribute to depression. A review of depression in late life found that the prevalence of depression in the elderly is not higher if factors associated with aging are controlled for.^31^

### Depressive symptoms and disability‐related factors

In our regression model with disability‐related factors, two factors were significantly associated with suspected depression: more self‐perceived impairments and less perceived participation. Of these factors, impairments, commonly perceived in people with LEoP, had the highest OR. This result contrasts somewhat to other studies of survivors of polio, reporting no association between motor function and depressive symptoms^9^ or between physical decrement and psychological well‐being.^4^ However, another study has reported more depressive symptoms in people with LEoP than in survivors of polio without LEoP,^21^ indicating that typical LEoP‐related impairments are indeed associated with depressive symptoms. The study by Kemp et al. also found that LEoP itself was not related to higher depression scores; instead the person's attitude toward his/her disability was more important.^21^ Because the SIPP scale assesses a variety of impairments including pain, cold intolerance, concentration difficulties, and fatigue,^16^ our results are somewhat difficult to compare with other studies. Although different aspects of impairments seem to have varying associations with depressive symptoms, it is important to consider them in clinical settings.

We also found that less perceived participation was significantly associated with suspected depression. This is in agreement with another study of older adults with a disability, where social participation such as visiting others, having an active leisure time and helping others were associated with fewer depressive symptoms.^11^ Other studies have also found that restrictions in activities of daily living are significantly associated with depressive symptoms.^32^, ^33^, ^34^ In people with LEoP, participation has been found to be a determinant of life satisfaction,^35^ which is in agreement with our results, as a high life satisfaction could be seen as an opposing anchor point to depression.^22^

Mobility, dichotomized as being able to walk 100 meters or not, was not associated with suspected depression. This could be interpreted as if you find ways to be mobile and maintain valued participation, your reduced walking ability, as a result of your prior polio, has a minor impact on the occurrence of depressive symptoms.

### Clinical implications and future research

The relatively high occurrence of depressive symptoms in people with LEoP implies that suspected depression might be underdiagnosed and that screening for depressive symptoms among these people is important. In particular, those who are single and those with more impairments and less perceived participation seem to be at risk. Awareness of depressive symptoms in people with LEoP and early interventions could prevent symptoms from evolving into a depression and improve mental health. Also, awareness of factors contributing to depression is important, and modifiable factors should be targeted in rehabilitation. If barriers due to the consequences of a disability are removed, people could maintain valued activities and social interactions, which may lead to improved mental health. Further studies of depressive symptoms and the impact of rehabilitation intervention targeting disability‐related factors are warranted.

### Strengths and limitations

A strength of the study is the use of valid and reliable rating scales. Limitations include the cross‐sectional design, not allowing us to make any causal conclusions, and the use of self‐reporting rating scales. Also, we did not include the use of mobility aids in the analyses as we had to restrict our independent variables in the logistic analyses. Reluctance to use mobility aids, or barriers to receive them, could have an impact on participation and make the people more prone to depression. Another limitation is the relatively small number of participants.

## CONCLUSION

The relatively high occurrence of suspected depression in people with LEoP implies that screening for depression is important. It remains to be determined if rehabilitation interventions targeting disability‐related factors can affect mental health in people with LEoP.

## FUNDING INFORMATION

The study received financial support from Stiftelsen för Bistånd åt Rörelsehindrade i Skåne, Norrbacka Eugenia Stiftelsen and Skåne Regional Council.

## DISCLOSURE

The other authors have no conflict of interest to disclose.
